# Apolipoprotein M Serum Levels Correlate with IgA Vasculitis and IgA Vasculitis Nephritis

**DOI:** 10.1155/2019/1825849

**Published:** 2019-12-11

**Authors:** Jiali Wu, Lagu He, Le Bai, Li Tan, Min Hu

**Affiliations:** Department of Laboratory Medicine, The Second Xiangya Hospital, Central South University, Changsha, Hunan 410011, China

## Abstract

**Objective:**

IgA vasculitis (lgAV) is the most frequent vessel vasculitis in children, and the prognosis is related to the children's age and degree of nephritis. This study is aimed at investigating serum apolipoprotein M (apoM) levels in patients with lgAV patients and at evaluating the association between apoM and disease severity.

**Methods:**

A total of 109 lgAV patients and 76 age- and sex-matched healthy controls were included. The age and gender of the study participants were matched. ApoM levels were measured by an enzyme-linked immunosorbent assay. Additionally, the serum levels of lipids, apolipoproteins, kidney biochemical profiles, immunoglobulins (IgA, IgG, IgM, and IgE), and the complements (C3 and C4) were assessed using an automatic biochemical analyzer.

**Results:**

ApoM was increased significantly in lgAV patients compared to healthy controls. ApoM, meanwhile, was lower in patients with nephritis than in those without nephritis. The apoM levels were higher in classes I and II IgA vasculitis nephritis (lgAVN) patients than in classes III and IV. Besides, the apoM serum level < 24.81 mg/L was an independent predictive factor for lgAVN and can be independently associated with the presence of nephritis in lgAV patients. Meanwhile, the serum apoM concentration negatively correlated with the ISKDC grading score in lgAVN patients.

**Conclusions:**

Serum apoM was elevated in lgAV patients and decreased gradually with the ISKDC grading score. ApoM (OR = 0.32, 95%CI = 0.12‐0.85, *p* = 0.023) was identified as a protective factor for nephritis in all lgAV patients.

## 1. Introduction

lgAV is the most common vessel vasculitides in children, which is self-limited. The incidence of lgAV has been increasing in recent years, which varies from 10 to 30 cases per 100,000 children younger than 17 years [1, 2]. It is characterized clinically by palpable purpura, arthrocele or arthralgia, gastrointestinal symptoms, and renal disease [3].

The prognosis is related to the children's age and degree of nephritis. The nephritis is called IgA vasculitis nephritis (lgAVN), which develops in 30%–50% of patients with lgAV [4]. LgAVN is potentially the most severe complication, and the prognosis is mostly dependent on the severity of nephritis [5, 6]. It was pointed out that 12.8% of lgAVN patients had an unfavourable outcome [4, 7].

Apolipoprotein M (apoM) is a 26 kD apolipoprotein that is a member of the lipocalin protein superfamily and is exclusively expressed in hepatocytes and kidney tubular cells [8, 9]. ApoM exerts a variety of biological functions, such as antioxidative function [10], anti-inflammatory function [11], promoting pre-*β* HDL formation [12], and increasing cholesterol efflux from foam cells [13]. Several studies reported that apoM plays important roles in diabetes mellitus (DM) [14, 15], endothelial inflammation [16], and coronary artery disease (CAD) [17].

However, the study of evaluating apoM in lgAV, an inflammatory disease of the small blood vessels, and its complication, lgAVN, has never been reported before. Therefore, the purpose of this study was to investigate whether apoM is changed in lgAVN patients and to evaluate its possible association with disease severity.

## 2. Methods

### 2.1. Subjects

The patients were recruited from the Second Xiangya Hospital between March 2017 and December 2017. One hundred eighty-five children were enrolled in this study, with 109 lgAV patients and 76 healthy children. The European League against Rheumatism and Pediatric Rheumatology European Society (EULAR/PReS) criteria was used to diagnose lgAV: palpable purpura must be present (mandatory criterion) in association with at least one of the following: arthritis or arthralgia, diffuse abdominal pain, any biopsy showing a predominant IgA deposition, and/or nephritis (hematuria and/or proteinuria). The lgAVN group comprised patients with lgAV showing nephritis (hematuria and/or proteinuria) [18]. The exclusion criteria for lgAV were as follows: (1) receiving any steroid or immunosuppressant treatments before blood samples were collected; (2) accompanied with diabetes, thrombocytopenic purpura, and liver or other kidney diseases. The histological grades of the renal biopsy were graded in accordance with the International Study of Kidney Disease in Children (ISKDC) classification [19] as follows: class I, minor glomerular abnormalities; class II, pure mesangial proliferation; class III, minor glomerular abnormalities or mesangial proliferation, with crescents/segmental lesions (sclerosis, adhesion, thrombosis, and necrosis) in <50% of the glomeruli; class IV, same as class III but with crescents/segmental lesions in 50–75% of the glomeruli; and class V, same as class III but with crescents/segmental lesions in >75% of the glomeruli; the vascular component was graded as follows: class 1, essentially normal; class 2, <25%; class 3, 25% to 50%; and class 4, >50% [20].

### 2.2. Blood Sampling

After an overnight fast and 30 min rest, blood samples were collected from each subject. Serum was obtained by centrifugation at 3500 rpm for 5 min, and aliquots were stored at -80°C for analysis.

### 2.3. Enzyme-Linked Immunosorbent Assay (ELISA) for apoM Assessment

Serum apoM levels were measured using a sandwich ELISA (Yuan Tai Bio Inc., Changsha, Hunan, China). Optical density (OD) was measured at 450 nm (with the background reading at 620 nm) on an ELX-800 absorbance reader (BioTek Instruments, Inc., Winooski, VT, USA). The concentration of apoM (as mg/L) in each sample was derived from a standard curve.

### 2.4. Laboratory Examination

Levels of serum blood urea nitrogen (BUN), creatinine (CRE), uric acid (UA), triglyceride (TG), total cholesterol (TC), HDL cholesterol (HDL-C), LDL cholesterol (LDL-C), apolipoprotein A (apoA), apolipoprotein B (apoB), and C-reactive protein (CRP) levels were determined using an ARCHITECTc8000 System (Abbott Laboratories, Irving, TX, USA). Levels of serum immunoglobulins (IgA, IgG, IgM, and IgE) and the complements (C3 and C4) were determined using Beckman IMMAGE800.

### 2.5. Statistical Analysis

Continuous data are presented as median (range), and categorical data are expressed as percentages. Continuous data were analyzed with the Student *t*-test or one-way analysis of variance (one-way ANOVA) for traits with the normal distribution. Pearson's correlation analysis was used to test for associations between apoM and variables with a normal distribution. The Spearman correlation analysis was used to analyze variables with skewed distributions. Binary logistic regression models were carried out to identify the risk factors for lgAVN patients. All analyses were performed with SPSS 20.0 (SPSS, USA) or GraphPad Prism 5.0 (GraphPad Software, La Jolla, CA, USA). Two-sided *p* < 0.05 was considered statistically significant.

## 3. Results

### 3.1. Clinical Characteristics of the Study Subjects

One hundred nine lgAV patients were included in the study, with 63 boys and 43 girls ranging in age from 1 to 15 (9.04 ± 0.71) years. Among the 109 lgAV patients, 43 patients developed nephritis (lgAVN). Among the 43 lgAVN patients, there are 25 patients with only purpura, 8 patients with purpura and arthritis, 8 patients with purpura and abdominal pain, and 2 patients with purpura, arthritis, and abdominal pain. Among the 66 lgAV patients, there are 31 patients with only purpura, 18 patients with purpura and arthritis, 12 patients with purpura and abdominal pain, and 5 patients with purpura, arthritis, and abdominal pain.

No statistical differences in age (7.88 ± 3.024, 9.42 ± 4.32, and 8.79 ± 4.04) and gender were observed among healthy controls, lgAVN, and lgAV without nephritis groups (all *p* > 0.05).

### 3.2. The Serum Lipid Levels and Apolipoprotein Levels in Healthy Controls, lgAVN, and lgAV without Nephritis Patients

The serum lipid levels in the three groups are shown in Table 1; compared with the healthy controls, lgAVN and lgAV without nephritis patients had significantly higher serum TC and LDL-C levels.

**Figure 1:**
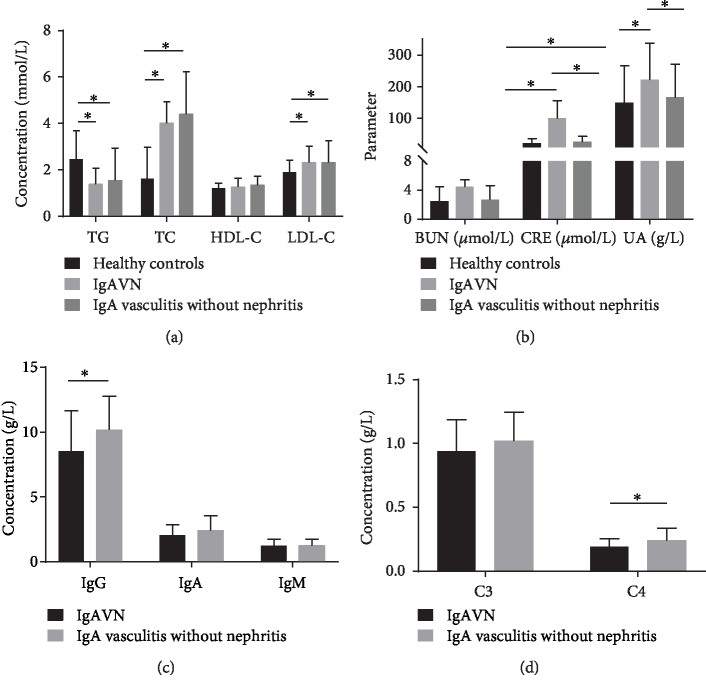
Serum concentrations of lipid levels, renal function markers and immunoglobulins, and complements in lgAV patients and healthy controls. (a) lgAV patients exhibit decreased serum TG levels and elevated serum levels of TC and LDL-C. (b) lgAV patients exhibit elevated serum CRE levels. (c) lgAV without nephritis patients have elevated lgG levels. (d) lgAV without nephritis patients have elevated C4 levels. ^∗^*p* < 0.05.

Meanwhile, there was no significant difference between the healthy controls, lgAVN patients, and lgAV without nephritis patients in average HDL-C serum levels or apoB serum levels (Figure 1(a)).

### 3.3. The Serum Kidney Biochemical Profile Levels in Healthy Controls, lgAVN Patients, and lgAV without Nephritis Patients

As shown in Table 1, the serum CRE levels of lgAV patients were significantly higher than those of the healthy subjects. Notably, the serum CRE and UA levels of the patients in the lgAVN group were even higher than those observed in the lgAV without nephritis group.

Meanwhile, there was no significant difference between the healthy controls, lgAVN patients, and lgAV without nephritis patients in average BUN serum levels (Figure 1(b)).

### 3.4. Comparison of Immune Function in lgAV without Nephritis Patients and lgAVN Patients

The serum lgG and C4 levels of the patients in the lgAV without nephritis group were even higher than those observed in the lgAVN group.

Meanwhile, there was no significant difference between lgAVN patients and lgAV without nephritis patients in average C3, lgM, lgA, or lgE levels (Figures 1(c) and 1(d)).

### 3.5. Serum apoM Levels Are Elevated in lgAV Patients

Kidney biopsy was performed on lgAVN patients. According to the ISKDC classification, 18 patients were in classes I and II, and 25 patients were in classes III and IV (13 in class IIIa, 7 in class IIIb, 5 in class IV).

**Figure 2:**
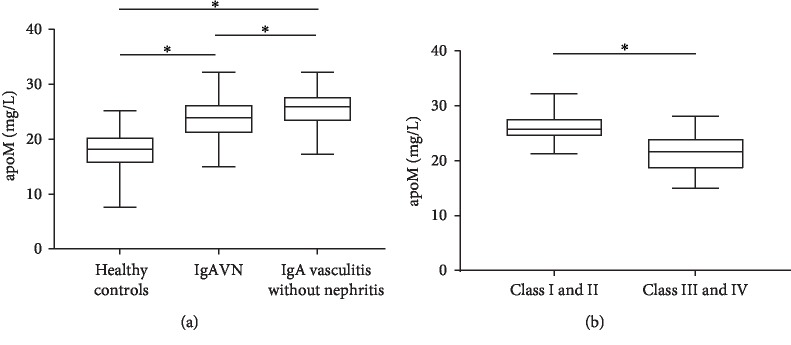
Serum apoM levels of lgAV patients and healthy controls. (a) apoM levels in lgAV patients were higher than those of healthy controls. (b) ApoM levels in IgA vasculitis nephritis patients with classes I and II were higher than those of patients with classes III and IV.

Serum apoM levels in lgAV patients were higher than values obtained for healthy controls. The most pronounced apoM increase in lgAVN patients was observed in classes III and IV (21.62 (19.16-23.7984) mg/L), followed by classes I and II (25.72 (24.58-27.3) mg/L, *p* < 0.05; Figure 2).

### 3.6. Association between Serum apoM and ISKDC in lgAVN Patients

As showed in Table 2, the serum apoM concentration negatively correlated with ISKDC in the lgAVN patients (*r* = −0.693, *p* < 0.001). Besides, ISKDC score negatively correlated with lgG in the lgAVN patients (*r* = −0.348, *p* = 0.022) (Table 2).

### 3.7. Predictive Factors for Nephritis

To identify the risk factors associated with lgAVN, a binary logistic regression model of the lgAV patients was built. In univariate analysis, apoM (OR = 0.339, 95%CI = 0.181‐0.877, *p* = 0.022) was identified as a protective risk factor. In contrast, CRE (OR = 3.753, 95%CI = 1.472‐9.572, *p* = 0.006) was found to be a risk factor for lgAVN onset (Table 3).

In multivariate analysis, apoM (OR = 0.37, 95%CI = 0.156‐0.877, *p* = 0.024) was identified as a protective factor.

## 4. Discussion

In this study, we measured the serum apoM levels in lgAV patients and healthy controls and screened for the association between serum apoM levels and ISKDC, a gold standard for severity assessment of renal. We found that apoM increased significantly in lgAV patients compared to healthy controls. ApoM, meanwhile, was lower in patients with nephritis than in those without nephritis. Also, the apoM serum level < 24.81 mg/L was an independent predictive factor for lgAVN and can be independently associated with the presence of nephritis in lgAV patients. Meanwhile, the serum apoM concentration negatively correlated with ISKDC in lgAVN patients.

It is reported that the pathogenesis of lgAV is mainly the humoral immunity mediated by lgA, and the systemic inflammatory response and tissue damage caused by the deposition of lgA on the wall of small blood vessels play an essential role in the pathogenesis of IgA vaculitis [21].

Several studies have been reported that apoM was reported in metabolic diseases, autoimmune diseases, and inflammatory diseases. These studies proved apoM could be used as a novel potential marker to evaluate disease activity in chronic obstructive pulmonary disease (COPD) [22] and systemic lupus erythematosus (SLE) [23], and recognized as a critical unfavourable prognostic determinant in COPD and SLE. Meanwhile, apoM is closely related to lipid metabolism. In primary nephrotic syndrome (PNS) [24], serum apoM level is negatively correlated with proteinuria.

In this study, lgAV patients exhibited lower serum levels of apoA and higher serum levels of LDL-C than the control subjects. In contrast, these patients also presented with higher serum levels of apoM than the controls.

It is reported that serum apoM has a positive correlation with serum HDL-C [10, 25]. This is particularly true for HDL-C, as approximately 96% of apoM is bound to HDL particles [26]. This may be since most of the apoM is anchored to the HDL. ApoM was shown to be exchanged from HDL-C to LDL-C because of altered HDL-C amounts under inflammatory conditions [27, 28]. These partly can explain why both LDL-C and apoM were increased in lgAV patients, although there was no correlation between them.

In this study, the apoM level was negatively correlated with ISKDC grading scoring, which is a gold standard for the severity assessment of renal. Meanwhile, in multivariate analysis, apoM was the independent predictor of the presence of nephritis in lgAV patients. Serum apoM levels in lgAVN patients were lower than lgAV without nephritis patients. The apoM serum level in the lgAVN patients of classes I and II was higher than the lgAVN patients of classes III and IV. Some reasons may account for this discrepancy. ApoM is exclusively expressed in kidney tubular cells. The systemic inflammatory response caused by the deposition of lgA causes elevated levels of apoM. The deposition of immune complex in lgAV patients causes the inflammatory reaction, and the expression of inflammatory factors that upregulate apoM increases, leading to an increase of apoM synthesis. Meanwhile, apoM loss was increased due to damage of renal tubular epithelial cells, and the content of apoM decreased with the aggravation of renal damage.

Due to the vital role of apoM in autoimmune disease, it is essential to understand the role of apoM concerning autoimmune diseases. Du et al. [23] reported that serum APOM levels were decreased in patients with SLE and correlated with disease activity. Hu et al. demonstrated that APOM promoter polymorphisms (rs805297 and rs805296) were significantly associated with RA [29]. Meanwhile, APOM C-1065A polymorphism is associated with an increased risk for developing rheumatoid arthritis and dyslipidemia in rheumatoid arthritis patients [30]. Furthermore, Wang et al. [31] showed that apoM could modify T lymphocyte subsets in the murine spleen. Indeed, the ApoM-S1P-S1P1 signalling axis restrains the lymphocyte compartment and, subsequently, adaptive immune responses [32]. Recent studies showed that ApoM-bound S1P reduces endothelial inflammation through sphingosine 1-phosphate receptor 1 (S1P1) [33, 34].

We proposed that apoM-S1P may play a role in resisting inflammation and immune dysfunction in lgAV patients. What is more, it may be used as a potential helpful agent in lgAV patients.

There are several limitations to our present study. First, the number of patients with lgAVN patients was relatively small. Further studies with a larger patient cohort are therefore needed to confirm our findings. Meanwhile, the results of 24 h urine volume and urine protein were challenging to obtain in children, and the results of cellular immunity and prognostic analysis were not obtained. These data would help determine whether apoM could be a serum marker to facilitate the management of lgAV in clinical practice or a potential helpful agent in lgAV treatments.

In general, our study found that the apoM level increased in lgAV patients. ApoM loss increased in lgAVN patients due to renal injury, resulting in a decrease in apoM serum level, and the apoM level decreased with the aggravation of the renal injury. The serum apoM level was negatively correlated with the ISKDC grading score in lgAVN patients.

## 5. Conclusion

The serum apoM level in patients with lgAV was found to be higher than that in healthy controls and negatively correlated with ISKDC grading score in lgAVN patients, reflecting apoM could be a marker of monitoring the progress of lgAVN.

## Figures and Tables

**Table 1:** Clinical and biochemical characteristics of the study subjects.

	Healthy control	lgAVN	lgAV without nephritis	*p*
Subjects, *n*	76	43	66	
Demographics				
Age	7.88 ± 3.024	9.42 ± 4.32	8.79 ± 4.04	>0.05
Gender (male/female)	44/32	26/17	37/29	>0.05
Lipid profiles				
TG (mmol/L)	2.75 (1.12-3.39)^b^	1.26 (0.8-1.66)^a^	1.08 (0.67-1.87)^a^	<0.05
TC (mmol/L)	0.84 (0.59-2.99)^b^	3.93 (3.44-4.56)^a^	3.98 (3.19-4.85)^a^	<0.05
HDL-C (mmol/L)	1.23 (1.05-1.37)	1.21 (1-1.36)	1.29 (1.03-1.55)	>0.05
LDL-C (mmol/L)	1.9 (1.5-2.16)^b^	2.21 (1.77-2.75)^a^	2.1 (1.57-2.78)^a^	<0.05
apoA (g/L)	1.06 (0.99-1.15)^b^	0.9 (0.75-1.04)^a^	0.93 (0.78-1.06)^a^	<0.05
apoB (g/L)	0.57 (0.47-0.66)	0.58 (0.49-0.7)	0.58 (0.45-0.72)	>0.05
apoM (mg/L)	18.17 (15.83-20.22)^a^	23.92 (21.25-26.1)^b^	25.96 (23.50-27.46)^c^	<0.05
Kidney biochemical profiles				
BUN (*μ*mol/L)	3.8 (3.25-4.47)	4.02 (2.86-5.5)	4.09 (2.99-4.95)	>0.05
CRE (*μ*mol/L)	30.9 (24.23-36.4)^a^	41.1 (36-53)^b^	35.55 (30.75-42.43)^c^	<0.05
UA (g/L)	230.05 (181.93-269.63)^a^	265 (218.6-330.3)^b^	223.3 (187.13-281.55)^a^	<0.05
hs-CRP (mg/L)	/	1.94 (1.02-3.87)	3.74 (1.37-12.1)	<0.05
Immunoglobulins				
lgG (g/L)	/	8.4 (6.16-11)	9.97 (8.29-11.7)	<0.05
lgM (g/L)	/	2 (154-2.35)	2.2 (1.52-3.19)	>0.05
lgA (g/L)	/	1.25 (0.84-1.42)	1.05 (0.87-1.56)	>0.05
lgE (g/L)	/	150.8 (59.09-447.3)	275.05 (119.38-298.63)	>0.05
Complements				
C3 (g/L)	/	0.95 (0.8-1.13)	1 (0.9-1.15)	>0.05
C4 (g/L)	/	0.18 (0.15-0.23)	0.23 (0.18-0.28)	<0.05

abc means not sharing a common superscript are significantly different among the healthy controls, lgAVN, and lgAV without nephritis groups at *p* < 0.05. Data are presented as median (range). TG: triglyceride; TC: total cholesterol; HDL-C: high-density lipoprotein cholesterol; LDL-C: low-density lipoprotein cholesterol; apoA1: apolipoprotein A1; apoB: apolipoprotein B; apoM: apolipoprotein M.

**Table 2:** Correlation between ISKDC and the serum apoM level.

	*R*	*p*
apoM	-0.693	<0.001
TG	0.222	0.152
TC	0.295	0.055
HDL-C	-0.005	0.975
LDL-C	0.209	0.055
apoA	-0.157	0.316
apoB	0.136	0.386
BUN	0.053	0.734
CRE	0.336	0.028
UA	0.133	0.395
lgG	-0.348	0.022
lgM	-0.161	0.302
lgA	0.091	0.562
lgE	0.077	0.624
C3	-0.015	0.926
C4	-0.01	0.951

**Table 3:** Predictive factors of nephritis in lgAV patients.

	OR	95% CI	*p*	OR	95% CI	*p*
TG	0.915	0.207-4.043	0.907	1.167	0.228-5.978	0.853
TC	1.44	0.652-3.183	0.368	2.892	0.694-12.058	0.145
HDL-C	0.738	0.339-1.609	0.445	0.622	0.23-1.684	0.35
LDL-C	1.295	0.599-2.803	0.511	0.594	0.169-2.087	0.416
apoA	0.745	0.344-1.612	0.455	0.925	0.379-2.255	0.864
apoB	0.834	0.382-1.822	0.649	0.685	0.261-1.797	0.442
apoM	0.339	0.181-0.877	0.022	0.37	0.156-0.877	0.024
BUN	1.385	0.633-3.033	0.415	1.002	0.411-2.542	0.962
CRE	3.753	1.472-9.572	0.006	2.774	0.934-8.06	0.066
UA	2.195	0.991-4.865	0.053	1.965	0.762-5.066	0.162

## Data Availability

All data generated or analyzed during this study are included in this published article.

## Abbreviations

apoA: Apolipoprotein A
apoB: Apolipoprotein B
apoM: Apolipoprotein M
HDL-C: High-density lipoprotein cholesterol
LDL-C: Low-density lipoprotein cholesterol
TG: Triglycerides
TC: Total cholesterol.
